# Disrupted resting-state attentional networks in T2DM patients

**DOI:** 10.1038/srep11148

**Published:** 2015-06-08

**Authors:** Wenqing Xia, Shaohua Wang, Hengyi Rao, Andrea M Spaeth, Pin Wang, Yue Yang, Rong Huang, Rongrong Cai, Haixia Sun

**Affiliations:** 1 Department of Endocrinology, affiliated ZhongDa Hospital of Southeast University, No. 87 Dingjiaqiao Road, Nanjing, China, 210009; 2 Medical school of Southeast University, No. 87 Dingjiaqiao Road, Nanjing, China, 210009; 3 Center for functional Neuroimaging, University of Pennsylvania, 3710 Hamilton Walk, Philadelphia, PA, USA, 19104; 4 Center for Sleep and Circadian Neurobiology, Perelman School of Medicine, University of Pennsylvania, 3710 Hamilton Walk, Philadelphia, PA, 19104.

## Abstract

Although Type 2 diabetes mellitus (T2DM) is a well-recognized risk factor for dementia, the neural mechanisms that underlie cognitive impairment in T2DM remain unclear. This study uses resting-state functional magnetic resonance imaging (fMRI) to examine attention network alterations in T2DM and their relationships to impaired cognitive performance. Data-driven independent component analysis was applied to resting-state fMRI data from 38 T2DM patients and 32 healthy controls to identify the dorsal attention network (DAN) and ventral attention network (VAN). Correlations were then determined among the resting-state functional connectivity (rsFC), clinical data, and neuropsychological scores. The T2DM patients exhibited decreased rsFC in the left middle frontal gyrus (MFG) and bilateral inferior parietal lobe (IPL) of the DAN, as well as the left IPL and right MFG/IFG of the VAN. In addition, the rsFC of the left MFG was inversely correlated with the Trail Making Test-B scores; the rsFC of the left IPL was positively correlated with the Digit Span Test scores but negatively correlated with HbA1c; and the rsFC in the right precuneus was positively associated with cognitive performance (without Bonferroni correction). In conclusion, T2DM affects resting-state attentional networks, which may be related to reduced attention and a hyperglycemic state.

Worldwide prevalence rates of type 2 diabetes mellitus (T2DM) and dementia are rapidly increasing and have become major global health challenges. An epidemiological study indicates an association between T2DM and cognitive decline^1^. In addition to memory deficits, some studies have shown that attention is compromised in patients with T2DM^2^,^3^. T2DM patients exhibited moderate decrements in attention compared with controls both at baseline and after a 4-year follow up^4^. We assume that a disruption of the attentional network may underlie the neurophysiological comorbidities of T2DM; however, the precise etiology of the attentional impairment in T2DM remains unclear.

Non-invasive neuroimaging techniques have been used to examine brain function changes in T2DM. Using positron emission tomography, one study identified an Alzheimer-like pattern of reduced regional cerebral glucose metabolism in frontal, parietotemporal, and cingulate regions in patients with prediabetes or early T2DM compared to normal controls^5^. Using resting-state blood oxygen level-dependent (BOLD) functional magnetic resonance imaging (fMRI), several studies have showed reduced functional connectivity between the nodes of the default mode network (DMN), such as the posterior cingulated cortex (PCC) and the hippocampus, in T2DM patients compared to normal controls^6^,^7^,^8^. These resting state functional connectivity (rsFC) studies used seed-based analysis, a hypothesis-driven method, to assess the temporal synchronization of neuronal activity between the seed and other remote areas. Therefore, the findings are limited to the connectivity pattern of the selected seed regions. Furthermore, previous studies have not focused on brain connectivity alteration in attentional networks and their relationships to attention deficits in T2DM.

Independent component analysis (ICA) is a data-driven method that automatically identifies meaningful brain networks and maps functional connectivity in the brain without the need for a priori defined seed regions^9^. Instead, ICA decomposes the entire resting-state BOLD data set into spatially distributed components that are maximally independent in a statistical sense^10^,^11^,^12^. Based on prior ICA-based resting-state fMRI investigations that examined different brain networks^13^,^14^,^15^,^16^, two networks related to attention have been well defined: The dorsal attention network (DAN) is comprised of the bilateral intraparietal sulcus and the frontal eye fields (FEF) and mediates top-down processing of attentional orientation. The ventral attention network (VAN) consists of the right lateralized temporal parietal junction (TPJ) and the ventral frontal cortex (VFC) and is involved in bottom-up processing of attentional re-orientation. Altered connectivity in the DAN and VAN have been demonstrated in different brain disorders with cognitive impairment, including Alzheimer’s disease (AD) and hepatic encephalopathy^16^,^17^.

In the present study, ICA was used in order to identify the DAN and VAN in a large sample of 70 adults, including 38 T2DM patients and 32 matched healthy controls. Relationships between attention network connectivity and clinical variables as well as between attention network connectivity and cognitive performance were assessed. We hypothesize that T2DM patients would exhibit reduced functional connectivity in the DAN and VAN and that functional connectivity within these networks would correlate with cognitive tests of attention.

## Material and methods

### Participants

This study was approved by the Research Ethics Committee of the Affiliated Zhongda Hospital of Southeast University. The methods were conduted in accordance with the approved guidelines. A total of 40 diabetic patients and 35 healthy subjects were recruited from clinics and hospitals after providing written informed consent. All participants were right-handed, were between 45 and 70 years of age, and had at least 6 years of education. All patients who met the diagnosis of T2DM based on the 1999 World Health Organization criteria^18^ and were on stable therapy with diet, exercise, insulin, or oral medications (The therapeutic agents used by the patients are provided in Supplementary table 1) were included. We considered one as having blood pressure lowering medications or cholesterol lowering medications if he/she had used related drugs in the past or currently. Patients with macrovascular diseases (e.g., cerebrovascular, or cardiovascular diseases) or clinically detectable microvascular complications (e.g., retinopathy, nephropathy, or neuropathy) were excluded. No patients complained of chronic pain or suffered symptoms of pain during scanning. Healthy controls matched for age, gender, BMI, and education level were recruited from the community. Individuals with a fasting glucose level > 5.6 mmol/l or a postprandial glucose level > 7.8 mmol/l were excluded.

Additional exclusion criteria included having a history of or current stroke, alcoholism, head injury, Parkinson’s disease, epilepsy, major depression or other neurological or psychiatric illnesses, a major medical illness (e.g., cancer, anemia or thyroid dysfunction), and severe visual or hearing loss.

### Clinical data and neuropsychological test information

Clinical diagnoses were conducted for all subjects after a baseline medical screening, a 50-minute neuropsychological battery, and a laboratory screening. Venous blood samples were obtained at 8 A.M. after an overnight fast of at least 10 hours. Each subject was then instructed to drink a 75 g glucose solution, and a second blood sample was collected 2 hours later. Fasting blood glucose, postprandial blood glucose, HbA1c, triglyceride, total cholesterol, low-density lipoprotein cholesterol (LDL-C), and high-density lipoprotein cholesterol (HDL-C) levels were assessed. Carotid artery ultrasound images were also obtained for each participant.

A battery of cognitive testing, including the Mini Mental State Exam (MMSE), Auditory Verbal Learning Test (AVLT), Rey-Osterrieth Complex Figure Test (CFT), Digit Symbol Substitution Test (DSST), Digit Span Test (DST), Trail Making Test-A and B (TMT-A and TMT-B), and Clock Drawing Test (CDT), was administered in a fixed order to evaluate general mental status, memory, attention, executive function, and visuospatial function. Because depression affects cognitive function and is more common in T2DM patients compared with control subjects^19^, the Hamilton Depression Scale was also administered by an experienced neuropsychiatrist under single-blind conditions.

### MRI acquisition and image preprocessing

Imaging data were acquired on a 3.0 T MRI scanner (Siemens MAGENETOM Trio). Cushions and earplugs were used to reduce head motion and scanner noise. Subjects were instructed to lie quietly with their eyes closed but avoid falling asleep, not to think of anything in particular, and to avoid head motion during fMRI. All participants were scanned at 11 A.M. on the same day that the clinical data were collected and the neuropsychological tests were conducted. Fluid-attenuated inversion recovery scans were also acquired: TR = 8,500 ms, TE = 94 ms, slices = 20, thickness = 5 mm, and voxel size 1.3 × 0.9 × 5 mm^3^. Functional images were collected axially using an echo-planar imaging (EPI) sequence with the following parameters: repetition time (TR) = 2000 ms, echo time (TE) = 25 ms, slices = 36, thickness = 4 mm, gap = 0 mm, field of view (FOV) = 240 × 240 mm^2^, acquisition matrix = 64 × 64, and flip angle (FA) = 90°. The resting-state fMRI (rs-fMRI) scan took 8 minutes and 6 seconds. High-resolution 3D T1-weighted axial images spanning the entire brain were acquired using the following parameters: TR = 1900 ms, TE = 2.48 ms, slices = 176, thickness = 1 mm, gap = 0 mm, FA = 90°, acquisition matrix = 256 × 256, and FOV = 250 × 250 mm^2^. The entire imaging protocol took 14 minutes and 23 seconds.

Quantitative assessments of the white matter hyperintensity (WMH) and lacunar infarcts were made on the fluid-attenuated inversion recovery images based on the age-related white matter changes scale^20^ with a single-blind method. Participants with a rating score > 1 were excluded.

Analyses were conducted using the Data Processing Assistant for rs-fMRI (DPARSF) program^21^, which is based on statistical parametric mapping (SPM8, http://www.fil.ion.ucl.ac.uk/spm) and rs-fMRI data analysis toolkits (REST, http://www.restfmri.net). Preprocessing was performed as previously described^22^. Briefly, 240 volumes were scanned, and the first 10 volumes were discarded, followed by a slice-timing adjustment, realignment for head-motion correction, and spatial normalization to the Montreal Neurological Institute (MNI) template (resampling voxel size = 3 × 3 × 3 mm^3^) in addition to smoothing with an isotropic Gaussian kernel (FWHM = 4 mm), detrend and filtering (0.01 − 0.08 Hz).

The data from 5 subjects (2 patients and 3 healthy controls) were excluded from the overall statistical analyses: 2 subjects were excluded for excessive motion (head movement exceeded 2.0 mm of the maximum translation in any of the x, y, and z directions or 2.0° of the maximum rotation about the three axes), 1 subject was excluded for poor quality images, and 2 subjects were excluded for a WMH rating score > 1.

### Independent component analysis

ICA was applied to the fMRI data using the groupICA (GICA) of the fMRI toolbox (http://www.nitrc.org/projects/cogicat/, MICA version beta 1.2)^23^. The fMRI toolbox reduced the data, applied the ICA algorithm and then performed back-reconstruction and converted the data into calibrated rsFC maps. In this study, we performed GICA 100 times and decomposed the dataset into 30 components. In these 30 maps, some maps reflect noise components whereas other maps reflect neuro-anatomical systems. According to previous studies^13^,^14^,^15^,^16^, the DAN is composed of the bilateral intraparietal sulcus and the FEF, whereas the VAN includes the right lateralized TPJ and the VFC. The independent components that best matched the DAN or VAN were identified in each group via visual inspection.

### Voxelwise-based gray matter volume correction

A voxel-based morphometry (VBM) approach was used to generate gray matter (GM) volume maps of all subjects. VBM was performed using the VBM8 toolbox (http://dbm.neuro.uni-jena.de/vbm) run in the Statistical Parametrical Mapping 8 (http://www.fil.ion.ucl.ac.uk/spm/) software on MATLAB 7.10.0. Briefly, cerebral tissues were segmented into gray matter, white matter, and cerebrospinal fluid using a unified segmentation algorithm. T1 magnetic resonance images were normalized to the Montreal Neurological Institute (MNI) template using affine linear registration, followed by Gaussian smoothing (FWHM = 8 mm, because the FWHM should be 2 to 3-fold the voxel size and T1 images have a relatively higher resolution). Finally, the resulting voxelwise GM volume maps were input as covariates during the functional data analysis.

### Statistical analysis

#### Demographic and clinical variable analysis

Analysis was performed using SPSS software (version 17.0). Differences in the demographic and cognitive data between the patients and healthy controls were analyzed using a *t* test for normally distributed variables, a nonparametric Mann-Whitney U test for asymmetrically distributed variables, and a χ^2^-test for categorical variables. All tests were conducted using a two-sided α-level of 0.05.

#### fMRI data analysis

The component maps for the participants were entered into a random-effect one-sample *t*-test to create a sample-specific component map (*p* < 0.05, after correcting for multiple comparisons using the False Discovery Rate, FDR). One-way ANOVA was used to compare differences in synchronization within the two attentional networks. All statistical analyses were performed with age, gender, education level, and BMI as nuisance covariates to control for potential influences. Monte Carlo simulations were performed using the AFNI AlphaSim program (http://afni.nih.gov/afni/docpdf/AlphaSim.pdf) (parameters: single voxel *p* value = 0.05, a minimum cluster size of 85 mm^3^, FWHM = 4 mm, and within a gray matter mask corresponding to the AAL atlas).

#### Correlation analysis

A ROI-based partial correlation analysis was calculated between the rsFC and the psychometric and clinical results. The mean Z values of each brain area that showed significant rsFC group difference were extracted using REST software (http://restingfmri.sourceforge.net/). Pearson’s correlation coefficients between a change in the rsFC strength and cognitive performance and clinical variables were subsequently analyzed using SPSS software (version 17.0), with a *p* value < 0.05 considered statistically significant. Age, gender, education level, BMI, hypercholesterolemia, intima-media thickness, and the presence of WMH and lacunar infarcts were entered as covariates. Bonferroni corrections were then used for multiple comparisons. Given the large age range of the study subjects, we also performed multiple regression in SPM8 to investigate the potential altered brain regions that accompanied increased age in each group as well as in the combined group.

## Results

### Clinical and neuropsychological data

The demographic characteristics and neuropsychological data for each group are presented in Table 1 and Table 2. No significant differences were observed between groups with respect to age, gender, education level, BMI, blood lipids, blood pressure, WMH, lacunar infarcts, intima-media thickness or medication. As expected, T2DM patients had increased HbA1c, fasting and postprandial glucose levels (all *p* < 0.001) compared with healthy controls. In terms of cognitive performance, T2DM patients had poorer scores for the CFT-copy, CFT-delay, DSST, DST, and TMT-B compared with the controls (*p* < 0.05).

### rsFC differences between patients and controls

**Figure 1:**
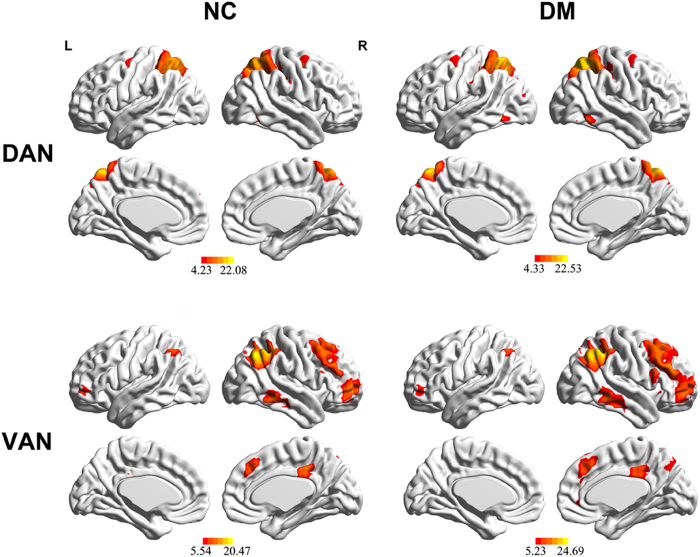
The typical anatomical pattern of the dorsal attention network (DAN) and ventral attention network (VAN) in healthy controls (NC) and type 2 diabetes mellitus (T2DM) patients (DM). Networks were identified using independent component analysis (*p* < 0.05, FDR corrected).

**Figure 2:**
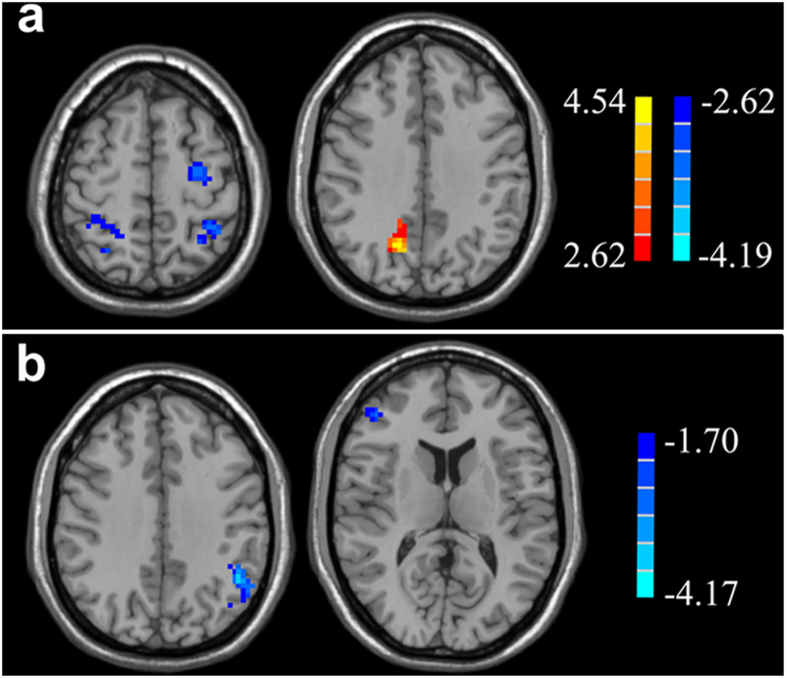
Significant decreases (cold color) and increases (warm color) in the resting-state functional connectivity of the DAN (**a**) and VAN (**b**) in T2DM patients. T2DM patients displayed decreased rsFC in the right inferior frontal gyrus (IFG), left middle frontal gyrus (MFG), and bilateral inferior parietal lobe (IPL) of the DAN, as well as the left IPL and right MFG/IFG of the VAN. Thresholds were set at a corrected *p* < 0.05, determined using a Monte Carlo simulation. Note that the left side corresponds to the right hemisphere.

The one-sample *t*-test revealed typical significant spatial patterns of the DAN and VAN in both the controls and T2DM patients (*p* < 0.05, corrected; Fig. 1). Fig. 2 displays the rsFC differences in the attentional related network according to two-sample *t*-tests be*t*ween groups. Compared with controls, T2DM patients displayed decreased rsFC in the left middle frontal gyrus (MFG) and bilateral inferior parietal lobe (IPL) of the DAN, as well as the left IPL and right MFG/IFG of the VAN (*p* < 0.05, corrected) (Table 3).

### Correlations between the rsFC of attentional networks and cognitive performance

**Figure 3:**
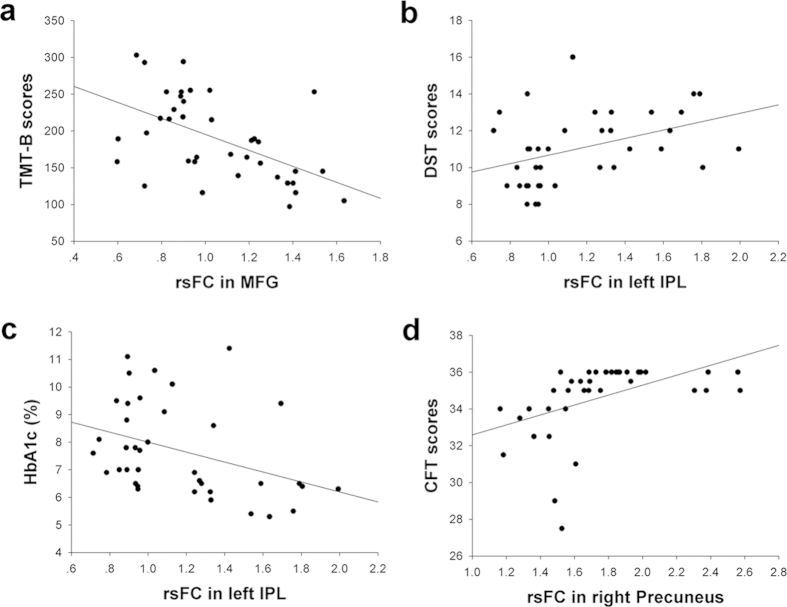
Correlations between the rsFC of specific regions in the DAN and the neuropsychological tests scores and HbA1c of T2DM patients. (**a**) A correlation between the rsFC of the left MFG and the TMT-B score (*r* = −0.533, *p* = 0.001). MFG, middle frontal gyrus; TMT -B, Trail Making Test-B. (**b**) A correlation between the rsFC of the left IPL and the DST score (*r* = 0.403, *p* = 0.012). IPL, inferior parietal lobe; DST, Digital Span Test. (**c**) A correlation between the rsFC of the left IPL and the HbA1c (*r* = −0.370, *p* = 0.022). (**d**) A correlation between the rsFC of the left precuneus and the CFT score (*r* = 0.473, *p* = 0.003). CFT, Rey-Osterrieth Complex Figure Test.

In T2DM patients, rsFC in the left MFG was inversely correlated with TMT-B scores (*r* = −0.533, *p* = 0.001; Fig. 3a), rsFC in the left IPL was positively correlated with DST scores but negatively correlated with the HbA1c (*r* = 0.403, *p* = 0.012 and *r* = −0.370, *p* = 0.022, respectively) (Fig. 3b,c). Additionally, a positive association (*r* = 0.473, *p* = 0.003) (Fig. 3d) between the CFT scores and the rsFC of the right precuneus was found. However, no significant results remained following a Bonferroni correction because of the many comparisons tested. No significant correlations were observed in the healthy control group. None of these correlations were significant (*p* < 0.05, corrected) in the VAN or the control group. We also did not detect any significant brain regions (*p* < 0.05, corrected) affected by an increase in age.

## Discussion

T2DM patients exhibit disrupted functional integration within attention-related brain networks, including the DAN and VAN, and disruptions in these networks significantly associated not only with neuropsychological scores but also with HbA1c.

Consistent with previous findings, patients with T2DM exhibited lower scores on all cognitive tests compared to control subjects; significant differences between groups were observed on tests of memory, attention, executive function, and visuospatial function. Notably, T2DM patients exhibit cognitive decrements in a variety of cognitive domains, which supports the presence of multidimensional cognitive abnormalities in T2DM patients^3^,^22^. Although only one test related to attention, namely DST, exhibited significant diversity between the groups, we believe that this result may be due to the sensitivity of the neuropsychological tests in this limited sample-size cohort.

In the present study the established DAN and VAN regions largely coincide with previously reported findings^13^,^14^,^15^,^16^. T2DM patients differed from healthy control subjects in the rsFC strength in these two attention networks. T2DM patients displayed both increased and decreased rsFC within the DAN, depending on the region, whereas, they also exhibited consistently decreased rsFC within regions of the VAN. T2DM patients displayed decreased rsFC within the IPL/SPL and MFG/IFG, which are core regions of these attention networks. A previous study that examined patients with AD found that these patients also exhibited decreased functional connectivity in the core regions of the DAN, including the IPL and SPL, bilateral IFG, left MFG and superior frontal gyrus (which covers the frontal eye-field)^24^. In the current study, fewer regions within the VAN displayed different levels of rsFC between patients and control subjects, and fewer significant correlations between the rsFC of regions in the VAN and cognitive performance were observed. Therefore, we suggest the VAN is stable and less susceptible to the impact of both diseases compared with the DAN. However, in contrast to AD in which several studies have suggested that left regions of the DAN are more seriously impaired than right regions^24^,^25^, we did not find evidence of lateralization in the impairment of attention networks. Taken together, although T2DM and AD appear to share a portion of brain pathogenesis^26^,^27^,^28^ that progress to cognitive impairment, T2DM-related attentional deficits may be not be comparable to the deficits present in AD patients. Therefore, the differences in cognitive impairment should be noted between the two disease states.

In addition to voluntary (top-down) orienting, the DAN also underlies other higher-level cognitive processes, such as visuospatial and executive function^29^. Consistent with these findings, we observed significant positive associations between TMT-B scores and z-values of the rsFC within the MFG and between DST scores and z-values of the rsFC within the left IPL. The TMT-B is a commonly used neuropsychological test that reflects frontal cortical functioning, and the DST is sensitive to attentional and working memory deficits. Our findings are consistent with previous studies showing associations between IPL activity and working memory performance (forward and backward digit span task^30^ and between DST performance and damage in the left hemisphere IPL^31^,^32^,^33^. These correlations between decreased rsFCs and poor performances in cognitive tests could potentially support the notion that changes in the DAN reflect neurobehavioral deficits in T2DM patients. Nevertheless, no other studies have found similar changes in this brain network or regions to demonstrate the deficits in attention to date. Several studies that have used cognitive tests with a larger sample size imply that T2DM patients have impaired attentional function. Thus, this issue remains to be confirmed by further studies.

In addition to these neuropsychological performance measures, HbA1c was also correlated with rsFC within the left IPL. Although previous research has demonstrated that HbA1c is negatively associated with improved attentional performance^34^, we did not identify a significant correlation between HbA1c and any of the neuropsychological tests. However, it is possible that changes in rs-fMRI within attention networks precede changes in behavioral tests and that rsFC in the IPL mediates attentional decline in T2DM patients. In-depth longitudinal studies are needed to confirm this speculation.

The precuneus plays a central role in visuospatial processing and execution, as well as in higher-order processes, such as voluntary attention shifts^35^,^36^. The CFT test is widely used to evaluate deficits in visuospatial constructional ability and executive function^37^, both of which are common consequences of attentional deficits. We observed a positive correlation between the rsFC within the right precuneus and CFT performance and therefore speculate that increased activity in the precuneus may act as a compensatory mechanism to avoid a decrease in the attentional ability of T2DM patients. These correlations were significant prior to multiple comparisons. When a Bonferroni correction was used, no significant correlations survived, which may, in part, because of the relatively strict calculation and limited sample size. Nevertheless, we believe that our research is still meaningful to provide directions for future investigations in this field. Another main negative result of this study is the undetectable potential effect of age. Although the effect of age on cognitive function has been proved^38^,^39^, it did not significantly influence the brain in the current cohort. Thus, a larger sample size and re-analyzing the data are necessary for future studies.

Previous fMRI studies have also identified differences in the brain between T2DM and healthy controls. For example, Musen and colleagues investigated brain abnormalities in T2DM patients using rs-fMRI^6^. They selected PCC as the ROI to produce meaningful results of altered functional connectivity between PCC and several brain areas, including the middle temporal gyrus, the inferior and medial frontal gyri, and the thalamus. Hoogenboom *et al.* combined diffusion tensor imaging and fMRI to identify the white matter alterations in T2DM patients that correlated with disrupted functional connectivity in the default-mode network^40^. In addition, to date, three data-driven studies have investigated the potential changes in the brain of T2DM: previous work from our laboratory used the amplitude of low frequency fluctuations (ALFF) method to investigate the neuronal activity of T2DM patients^22^, Wang *et al.*^41^ used the same method to further investigate the effects of diabetic vascular disease on the brain, and Cui *et al.*^42^ combined the ALFF with regional homogeneity (ReHo) methods and also observed alterations in the diabetic brain. ALFF and ReHo of the BOLD signal are thought to be indicators of the intensity and temporal synchronization of regional spontaneous neuronal activity in the whole brain^43^,^44^, whereas functional connectivity analysis describes spatiotemporal correlations between spatially distinct brain regions, as well as shows the correlations of voxel signals with the signal of a particular component^45^,^46^; higher functional connectivity between certain regions represents a higher degree of involvement of a particular network^47^. These discrepancies also lead to the diversity of results between the current study and previous findings.

Some limitations of our study should be noted. First, considering the current cross-sectional design and limited sample size with potential medication effects in the patients, further studies should try to examine a larger population receiving similar therapies and use a longitudinal approach to verify these findings. Second, we did not measure the intelligence quotient (IQ), which could drive differences in rsFC and cognitive tests. Finally, we only analyzed the rsFC within attentional related networks; however, other brain networks may also be affected by disease status and attentional function during the resting state, such as networks involved in executive function or working memory, which have been confirmed as prominent cognitive impairments in T2DM patients. If positive results are observed, in-depth studies should assess directional connectivity among these various resting-state networks. Conversely, if other networks are not altered, this finding would support the specificity of the current results.

In conclusion, for the first time, we used a data-driven approach to observe the functional connectivity changes within the attentional network in T2DM patients and found a significant disruption of attentional networks in these patients. Several core regions in the DAN, especially the IPL, likely underlie the attentional deficits in T2DM patients. Our data may potentially support rs-fMRI as a promising tool to detect attentional changes in T2DM and provide new insights into the neural deficits present in T2DM patients.

## Additional Information

**How to cite this article**: Xia, W. *et al.* Disrupted resting-state attentional networks in T2DM patients. *Sci. Rep.* **5**, 11148; doi: 10.1038/srep11148 (2015).

## Supplementary Material

Supplementary Information

## Figures and Tables

**Table 1:** Demographic and clinical characteristics.

Items	T2DM patients (n = 38)	Healthy controls (n = 32)	*p*-value
Age, years	58.6 ± 8.2	55.6 ± 7.1	0.108
Gender, female (%)	17(45)	15(47)	0.858
Education levels, years	9.7 ± 3.4	10.4 ± 2.0	0.338
Diabetes duration, years	9.9 ± 5.7	–	–
History of smoking (%)	9(24)	8(25)	0.898
BMI, kg/m^2^	25.0 ± 2.7	24.3 ± 2.4	0.267
Systolic BP, mmHg	131.6 ± 15.5	129.8 ± 14.3	0.601
Diastolic BP, mmHg	79.3 ± 8.6	80.8 ± 7.3	0.441
Hb_A1c_ , % (mmol/mol)	7.7 ± 1.7(61.0 ± 18.6)	5.1 ± 0.5(32.0 ± 5.5)	<0.001*
Fasting glucose, mmol/L	8.0 ± 2.3	5.2 ± 0.3	<0.001*
Postprandial glucose, mmol/L	11.8 ± 4.6	6.9 ± 0.8	<0.001*
Triglyceride, mmol/l	1.5 ± 0.7	1.4 ± 0.7	0.858
Total cholesterol, mmol/l	5.5 ± 1.1	5.7 ± 0.8	0. 604
LDL-C, mmol/l	3.4 ± 0.8	3.4 ± 0.5	0.862
HDL-C, mmol/l	1.4 ± 0.3	1.4 ± 0.3	0.699
White matter hyperintensity	0(0–6)	0(0–5)	0.182
Lacunar infarcts (%)	6(15)	4(12.5)	0.695
Intima-media thickness, mm	1.0 ± 0.2	1.0 ± 0.2	0.162
Blood pressure lowering medications (%)	16(42)	11(34)	0.508
Cholesterol lowering medications (%)	10(26)	4(13)	0.150

Values are mean±standard deviation, n(%), or median (range) . *P < 0.05 was considered significant.

Abbreviations: LDL-C, low-density lipoprotein cholesterol; HDL-C, high-density lipoprotein cholesterol.

**Table 2:** Cognitive scores and depressive symptoms.

Items	T2DM patients (n = 38)	Healthy controls (n = 32)	*p*-value
**General mental status**			
MMSE	28.8 ± 1.1	29.1 ± 1.2	0.259
**Memory**			
AVLT- total	32.1 ± 8.3	35.0 ± 5.4	0.092
AVLT-delay	6.2 ± 2.5	6.8 ± 1.7	0.246
CFT-delay	13.3 ± 6.1	19.0 ± 6.3	<0.001*
**Attention**			
TMT-A	72.5 ± 23.4	66.6 ± 17.0	0.231
DSST	43.1 ± 8.5	46.0 ± 8.2	0.154
DST	11.1 ± 2.0	12.3 ± 2.2	0.012*
**Executive function**			
TMT-B	189.5 ± 56.6	163.4 ± 50.4	0.048*
**Visuospatial function**			
CDT	3.3 ± 0.8	3.6 ± 0.5	0.070
CFT-copy	34.6 ± 2.0	35.6 ± 0.7	0.008*
**Depression**			
HAMD	1.1 ± 1.1	1.3 ± 1.0	0.501

Values are mean ± standard deviation. *P < 0.05 was considered significant.

Abbreviations: MMSE, Mini Mental State Exam; AVLT, Auditory Verbal Learning test; CFT, Rey-Osterreith Complex Figure Test; TMT, Trail Making Test; DSST, Digit Symbol Substitution Test; DST, Digit Span Test; CDT, Clock Drawing Test; HAMD, Hamilton Depression Scale.

**Table 3:** Regions showing significant differences in functional connectivity within attention-related brain networks between patients and healthy controls.

Brain regions	MNI Coordinates x, y, z (mm)	Peak t score	Voxels (mm^3^)
**Within DAN**			
L Middle Frontal Gyrus	−27, −9, 57	−3.0241	114
L Inferior Parietal Lobule	18, −48, 72	−3.1839	121
R Superior/Inferior Parietal Lobule	63, −27, 21	−3.5829	97
R Precuneus	15, −63, 36	4.5389	286
**Within VAN**			
L Inferior Parietal Lobule	−48, −51, 36	−3.3803	90
R Middle/ Inferior Frontal Gyrus	39, 51, −12	−2.7926	141

A corrected threshold of *p* < 0.05 determined by Monte Carlo simulation was taken as meaning that there was a significant difference between groups.

Abbreviations: DAN, dorsal attention network; VAN, ventral attention network; MNI: Montreal Neurological Institute; L, left; R, right.
