# Supplementary material for: Disrupted resting-state attentional networks in T2DM patients
Source: Sci Rep. 2015 Jun 8;5:11148. doi: 10.1038/srep11148 (PMC4459168; doi:10.1038/srep11148)
Supplement: Supplementary Information [file srep11148-s1.pdf]

Title page

Disrupted resting-state attentional networks in T2DM patients

Authors:

Wenqing Xia[1,2,#], Shaohua Wang[3,#,*] , Hengyi Rao[2], Andrea M Spaeth[4], Pin Wang[1], Yue Yang[1], Rong Huang[1], Rongrong Cai[1], Haixia Sun[1]

[#] These authors contributed equally to the preparation of this manuscript.

Author Affiliations:

[1] Medical school of Southeast University, No.87 Dingjiaqiao Road, Nanjing, China, 210009;

[2] Center for functional Neuroimaging, University of Pennsylvania, 3710 Hamilton Walk, Philadelphia, PA, USA, 19104;

[3] Department of Endocrinology, affiliated ZhongDa Hospital of Southeast University, No.87 Dingjiaqiao Road, Nanjing, China, 210009;

[4] Center for Sleep and Circadian Neurobiology, Perelman School of Medicine, University of Pennsylvania, 3710 Hamilton Walk, Philadelphia, PA, 19104

***Corresponding Author:**

Shaohua Wang, Ph.D, Prof.

Department of Endocrinology, affiliated Zhongda Hospital of Southeast University

No.87 Dingjiaqiao Road, Nanjing, China, 210009

E-mail: gyjwsh@126.com; Tel: 0086-25-83272261.

Supplementary Table 1. Therapeutic agents of the T2DM patients.

Therapeutic agents	Frequency (n)
Insulin	15
Biguanides	16
Glucosidase Inhibitors	6
Sulfonylureas	10
Non-Sulfonylurea	4
Thiazolidinediones	6
Only Diet and Exercise	1
